# The Differential Metabolic Response of Oral Squamous Cell Carcinoma Cells and Normal Oral Epithelial Cells to Cisplatin Exposure

**DOI:** 10.3390/metabo12050389

**Published:** 2022-04-25

**Authors:** Xun Chen, Sufang Kuang, Yi He, Hongyu Li, Chen Yi, Yiming Li, Chao Wang, Guanhui Chen, Shangwu Chen, Dongsheng Yu

**Affiliations:** 1 Hospital of Stomatology, Guangdong Provincial Key Laboratory of Stomatology, Guanghua School of Stomatology, Sun Yat-sen University, Guangzhou 510055, China; chenx598@mail2.sysu.edu.cn (X.C.); heyi33@mail2.sysu.edu.cn (Y.H.); lihongy5@mail2.sysu.edu.cn (H.L.); yich5@mail2.sysu.edu.cn (C.Y.); liym39@mail2.sysu.edu.cn (Y.L.); wangch283@mail2.sysu.edu.cn (C.W.); chengh86@mail.sysu.edu.cn (G.C.); 2 Center for Proteomics and Metabolomics, State Key Laboratory of Biocontrol, Guangdong Province Key Laboratory for Pharmaceutical Functional Genes, School of Life Sciences, Sun Yat-sen University, Guangzhou 510006, China; sufangkuang@126.com; 3 Guangdong Key Laboratory of Pharmaceutical Functional Genes, State Key Laboratory for Biocontrol, Department of Biochemistry, School of Life Sciences, Sun Yat-sen University, Guangzhou 510275, China

**Keywords:** metabolic response, metabolomics, cisplatin exposure, oral squamous cell carcinoma cells, normal oral epithelial cells

## Abstract

Metabolic reprogramming is one of the hallmarks of a tumor. It not only promotes the development and progression of tumor but also contributes to the resistance of tumor cells to chemotherapeutics. The difference in the metabolism between drug-resistant and sensitive tumor cells indicates that drug-resistant tumor cells have experienced metabolic adaptation. The metabolic response induced by chemotherapy is dynamic, but the early metabolic response of tumor cells to anticancer drugs and the effect of an initial response on the development of drug resistance have not been well studied. Early metabolic intervention may prevent or slow down the development of drug resistance. The differential metabolic responses of normal cells and tumor cells to drugs are unclear. The specific metabolites or metabolic pathways of tumor cells to chemotherapeutic drugs can be used as the target of metabolic intervention in tumor therapy. In this study, we used comparative metabolomics to analyze the differential metabolic responses of oral cancer cells and normal oral epithelial cells to short-term cisplatin exposure, and to identify the marker metabolites of early response in oral cancer cells. Oral cancer cells showed a dynamic metabolic response to cisplatin. Seven and five metabolites were identified as specific response markers to cisplatin exposure in oral cancer cell SCC-9 and normal oral epithelial cell HOEC, respectively. Glyoxylate and dicarboxylate metabolism and fructose, malate, serine, alanine, sorbose and glutamate were considered as specific enriched metabolic pathways and biomarkers of SCC-9 cells in response to cisplatin, respectively. The existence of differential metabolic responses lays a foundation for tumor chemotherapy combined with metabolic intervention.

## 1. Introduction

Tumor cells undergo metabolic reprogramming to produce enough energy and intermediates to meet the needs of rapid growth [1]. Metabolic reprogramming is actually a kind of metabolic adaptation, which dysregulates the expression and the activity of some metabolic enzymes, and then it affects the flux of metabolic pathways and the cell phenotype [2]. Metabolic reprogramming not only promotes the development and the progression of tumor [3,4] but also contributes to the resistance of tumor cell to chemotherapeutics. There is increasing evidence that cellular metabolism in tumors may be reprogrammed in response to drug treatment [5,6,7,8].

Cisplatin can induce DNA damage-mediated cell apoptosis and reactive oxygen species (ROS)-mediated oxidative stress [9,10,11]. It is commonly used in cancer treatment, including head and neck squamous cell carcinoma (HNSCC). Continuous exposure to cisplatin can induce drug resistance, thus limiting the efficacy of the drug. Although individual responses to cisplatin vary widely, most people develop resistance to cisplatin [12]. Acquisition of drug resistance is a complex process, involving both general and drug-specific mechanisms [2]. Metabolic adaptation is considered to be one of the mechanisms of cisplatin resistance in tumor cells.

By comparing the metabolic changes of cisplatin-resistant and sensitive tumor cells, it is revealed that cisplatin-resistant tumor cells may undergo metabolic adaptation in many aspects. Because cisplatin can induce ROS-mediated oxidative stress, cisplatin resistant cells first experienced the adaptation to oxidative stress. Cisplatin-resistant lung cancer cells increased ROS production [5,6], and high levels of ROS promoted epithelial-mesenchymal transition (EMT) [6]. These cells enhanced the uptake of glutamine, which can be converted into glutamate for the synthesis of glutathione thereby providing redox capacity to reduce the level of ROS. Thus, cisplatin resistant cells were highly sensitive to glutamine deprivation, and they were selectively killed by blocking glutamate flux [5]. Cisplatin-resistant ovarian cancer cells had a higher intracellular glutathione level than cisplatin-sensitive counterparts [7]. In cisplatin-resistant lung cancer cells, four glutathione-metabolizing enzymes, aminopeptidase N (CD13), glutathione peroxidase 4 (GPX4), 5-oxoprolinase (OPLAH) and ribonucleotide reductase regulatory TP53 inducible subunit M2B (RRM2B) were up-regulated, suggesting that glutathione metabolism plays a key role in acquiring cisplatin resistance [13]. It should be mentioned here that in addition to cisplatin, other antitumor agents, such as tyrosine kinase inhibitors, can also induce oxidative stress adaptation [14,15]. For example, Sorafenib is a tyrosine kinase inhibitor. GSH synthesis was enhanced in sorafenib-resistant leukemia cells, indicating that the resistant cancer cells experienced specific metabolic and redox adaptations [2,14].

In addition to oxidative stress adaptation, cisplatin-resistant tumor cells also have adaptive changes in material and energy metabolisms. Rewiring of lipid metabolism was observed in cisplatin-resistant ovarian cancer [7], bladder cancer [16], and lung cancer [17] cells. Compared with cisplatin-sensitive counterparts, the metabolism of amino acids was up-regulated in cisplatin-resistant neuroblastoma cells. Amino acid deprivation reduced cell survival and cisplatin-insensitivity. Treatment with cisplatin and hydroxychloroquine, a terminal autophagy inhibitor that blocks amino acid uptake abrogated amino acid metabolism in cisplatin resistant cells and it sensitized neuroblastoma cells to sub-lethal doses of cisplatin [18]. Cancer cells also undergo bioenergetic adaptation to meet the challenge of cisplatin. The metabolism of cisplatin-resistant ovarian cancer cells can switch between oxidative phosphorylation and glycolysis [8].

Although it is known that the metabolism of drug-resistant tumor cells has changed, the initial metabolic response of tumor cells to anticancer drugs and the effect of initial response on the development of drug resistance have not been well studied. The metabolic response induced by chemotherapy is dynamic [19], and the early metabolic response of cancer cells to drug stimulation should be different from that observed in drug-resistant cells. Early metabolic intervention may prevent or slow down the development of drug resistance. In addition, the difference in the metabolic response of normal cells to drugs is unclear. Compared with normal cells, the specific metabolites or metabolic pathways of tumor cells to chemotherapeutic drugs can be used as the target of metabolic intervention in tumor therapy. Metabolomics profiling can characterize the metabolic response of tumor cells to various chemotherapeutic agents. Metabolic reprogramming has been confirmed in several cisplatin-resistant cancer cells, such as ovarian cancer [7], lung cancer [5] and HNSCC [12]. In our previous study, we found metabolic reprogramming in oral squamous cell carcinoma cells [20]. In the current study, HNSCC cells were used as an example to explore their metabolic response to cisplatin. Comparative metabolomics was used to analyze the differential metabolic response of oral cancer cells and normal oral epithelial cells to short-term cisplatin treatment, and to determine the specific metabolites of early response in oral cancer cells.

## 2. Results

### 2.1. Oral Cancer Cells Showed a Dynamic Metabolic Response to Cisplatin

**Figure 1:**
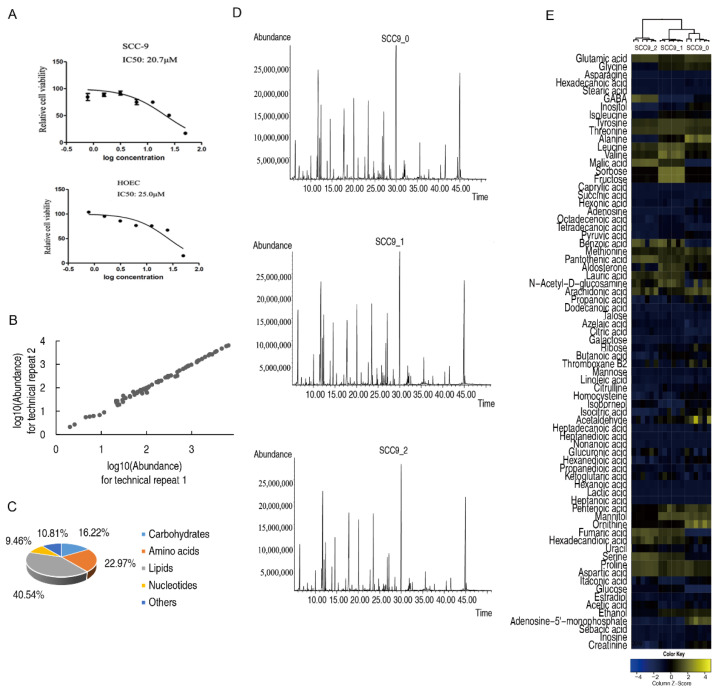
Metabolomics profiles of SCC-9 cells. Measurements of IC_50_ of SCC-9 and HOEC cells (**A**). Reproducibility of the metabolomics profiling platform (**B**). Classification of metabolites (**C**). Representative total ion current chromatograms (**D**). Cluster analysis of all metabolites (**E**). SCC9_0, SCC9_1 and SCC9_2 represent the SCC-9 cells treated with cisplatin for 0 h, 24 h or 48 h.

In order to obtain the optimal drug effect and the minimal cell death for metabolomics study, we first measured the IC_50_ of SCC-9 and HOEC cells to cisplatin, which are 20.7 μM and 25.0 μM, respectively (Figure 1A). Accordingly, both cells were treated with 10 μM cisplatin for 24 h or 48 h, and their metabolomes were profiled by GC-MS. The coefficient of technical repeats for GC-MS was between 0.990 and 0.999 (Figure 1B), indicating an excellent reproducibility. A total of 74 metabolites were identified, of which carbohydrates, amino acids, nucleotides, lipids, and other metabolites accounted for 16.22%, 22.97%, 40.54%, 9.46%, and 10.81%, respectively (Figure 1C). In SCC-9 cells, the total ion current chromatograms of metabolites showed that the peak pattern of cisplatin-treated samples was consistent with that of untreated samples, and the abundance of some metabolites was different (Figure 1D). The cluster analysis of all metabolites showed that each group clustered separately and they had good intra group reproducibility (Figure 1E). The 48 h treatment group was separated from the untreated group and the 24 h treatment group, which means that the metabolome of the 48 h treatment group showed greater changes compared with that in 24 h treatment group.

### 2.2. Effect of Cisplatin on Metabolome of Oral Squamous Cell Carcinoma Cells

**Figure 2:**
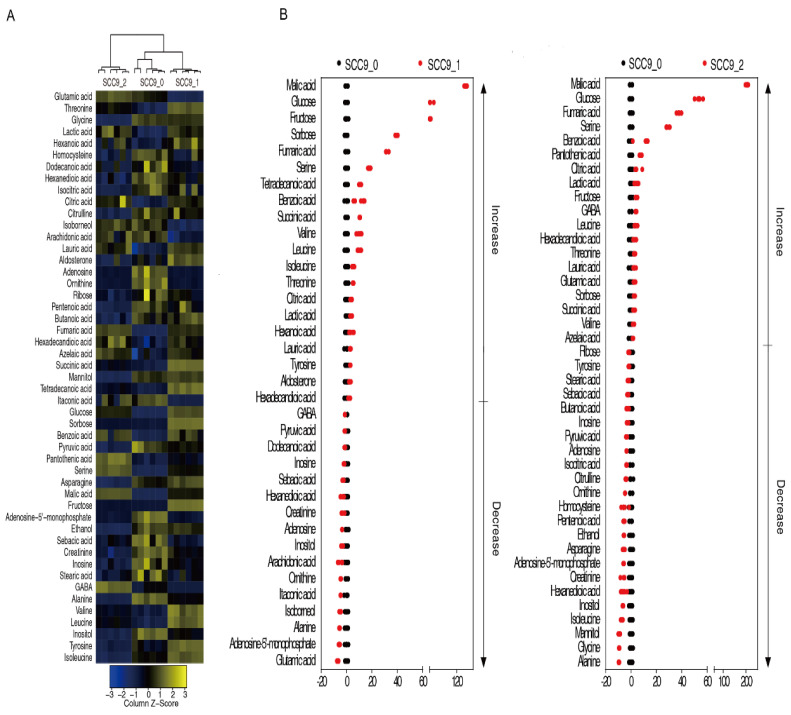
Differential metabolome of SCC-9 cells in response to cisplatin. Cluster analysis of the differential metabolites (**A**). The Z value distribution map of the differential metabolites, with untreated group as the control (**B**). SCC9_0, SCC9_1 and SCC9_2 represent the SCC-9 cells treated with cisplatin for 0 h, 24 h or 48 h.

**Figure 3:**
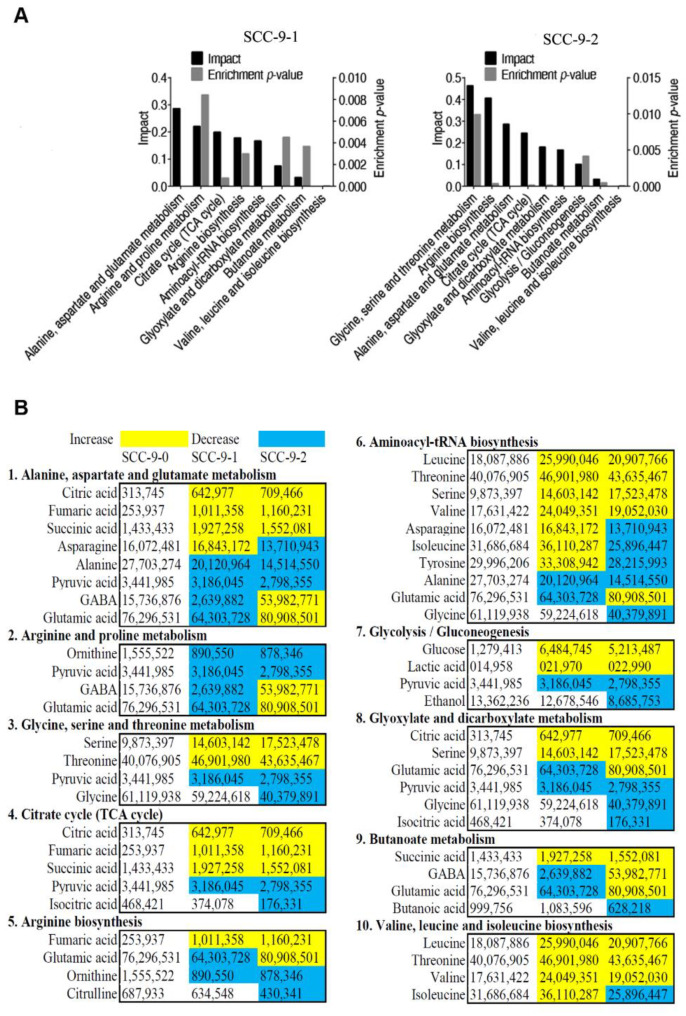
Pathway enrichment analysis of differential metabolites in SCC-9 cells treated with cisplatin. Significantly enriched metabolic pathways (**A**). Abundance of metabolites in the enriched metabolic pathways (**B**). SCC9_0, SCC9_1 and SCC9_2 represent the SCC-9 cells treated with cisplatin for 0 h, 24 h or 48 h.

The abundances of all metabolites were analyzed by the Kruskal–Wallis test. After 24 h or 48 h of cisplatin treatment, 36 or 42 differential metabolites were identified in SCC-9 cells (*p* < 0.01). Cluster analysis of the differential metabolites indicated that SCC-9 cells treated with cisplatin for 48 h clustered into a group, while cells treated with cisplatin for 24 h and untreated cells clustered into another group, supporting greater metabolic changes after 48 h of treatment (Figure 2A). The Z-value diagram showed that SCC-9 cells treated with cisplatin for 24 h or 48 h shared 15 up-regulated and 10 down-regulated differential metabolites compared with SCC-9 cells without drug treatment (Figure 2B) (Table 1). The pathway enrichment analysis of differential metabolites was carried out through the Metaboanalyst 4.0 online platform, and the *p* value was 0.01. The order of metabolic pathways ranked from high to low according to the impact value. The differential metabolites of SCC-9 cells treated with cisplatin for 24 h or 48 h were enriched in 8 or 9 metabolic pathways, respectively (Figure 3A). The three most important metabolic pathways enriched at each time point are alanine, aspartate, and glutamate metabolism; arginine and proline metabolism; citrate cycle or glycine, serine and threonine metabolism; arginine biosynthesis; and alanine, aspartate and glutamate metabolism, respectively. The cells treated for 24 h or 48 h shared 7 enriched metabolic pathways, including alanine, aspartate, and glutamate metabolism; citrate cycle; arginine biosynthesis; aminoacyl-tRNA biosynthesis; glyoxylate and dicarboxylate metabolism; butanoate metabolism; and valine, leucine, and isoleucine biosynthesis. The relative abundance of differential metabolites in the enriched metabolic pathway was shown in Figure 3B.

### 2.3. Marker Metabolites of Cisplatin Induced Metabolic Response in SCC-9 Cells

**Figure 4:**
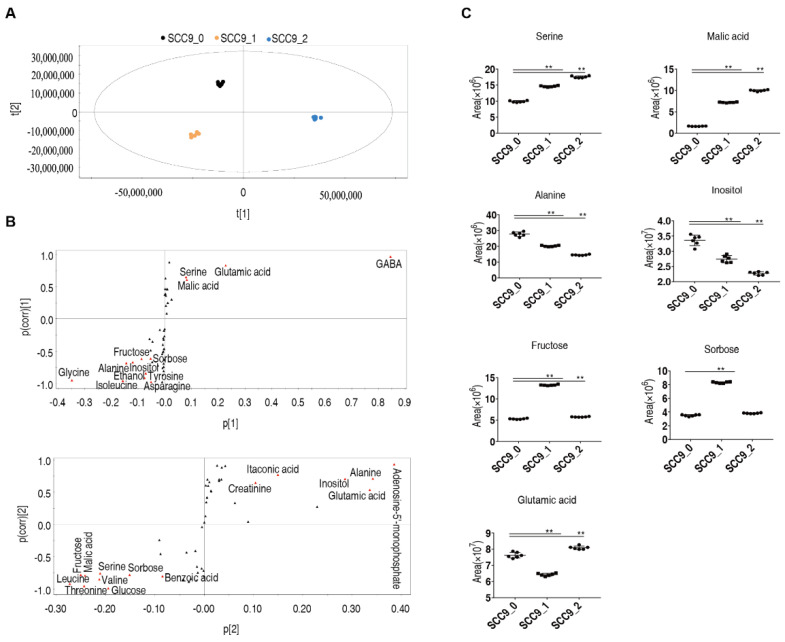
Screening of marker metabolites in SCC-9 cells stimulated by cisplatin. OPLS-DA analysis (**A**). S-plot analysis (**B**). Relative abundance of marker metabolites (**C**). SCC9_0, SCC9_1 and SCC9_2 represent the SCC-9 cells treated with cisplatin for 0 h, 24 h or 48 h. **, *p* < 0.01.

When the abundance of differential metabolites was analyzed by OPLS-DA, the 48 h treated group could be distinguished from the untreated and the 24 h treated groups by the t [1], and the untreated group was separated from the 24 h and 48 h treated groups by t [2] (Figure 4A). S-plot analysis was used to determine the marker metabolites in cisplatin induced metabolic response, with weight coefficient <−0.05 or >0.05 and correlation coefficient < −0.5 or >0.5. Thirteen marker metabolites were identified by t [1], and 15 metabolites by t [2] (Figure 4B). Seven common marker metabolites, fructose, malate, serine, inositol, alanine, sorbose, and glutamate, were identified by two factors (Figure 4C).

### 2.4. Metabolic Changes and Marker Metabolites of HOEC Cells Stimulated by Cisplatin

**Figure 5:**
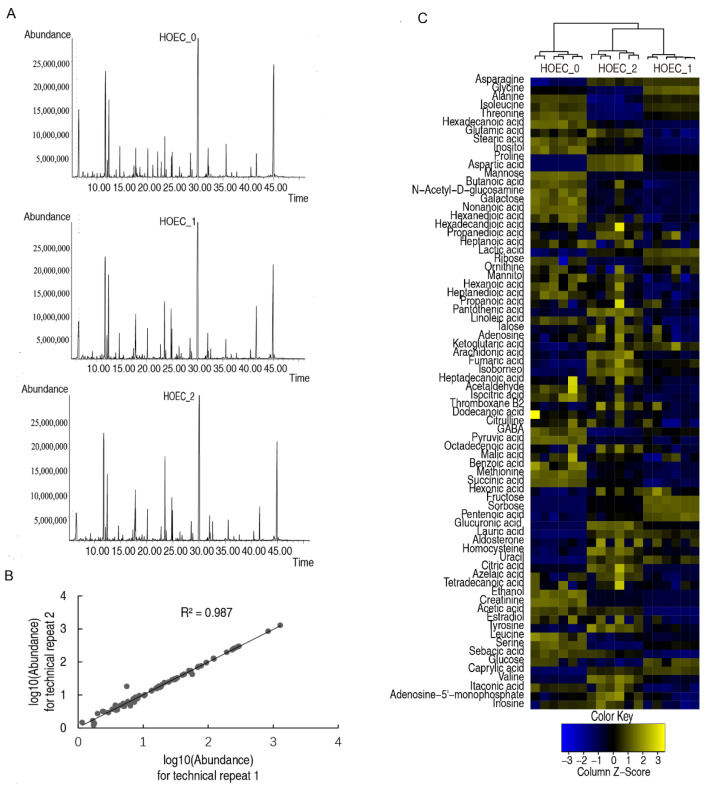
Metabolomics profiles of HOEC cells. Representative total ion current chromatograms (**A**). Reproducibility of the metabolomics profiling platform (**B**). Cluster analysis of all metabolites (**C**). HOEC_0, HOEC_1 and HOEC_2 represent the HOEC cells treated with cisplatin for 0 h, 24 h or 48 h.

**Figure 6:**
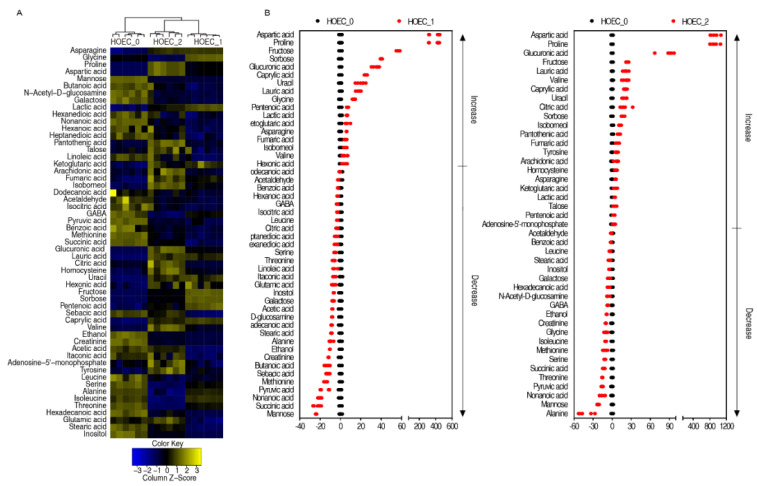
Differential metabolites of HOEC cells after cisplatin treatment. Cluster analysis of the differential metabolites (**A**). The Z value distribution map of the differential metabolites (**B**). HOEC_0, HOEC_1 and HOEC_2 represent the HOEC cells treated with cisplatin for 0 h, 24 h or 48 h.

**Figure 7:**
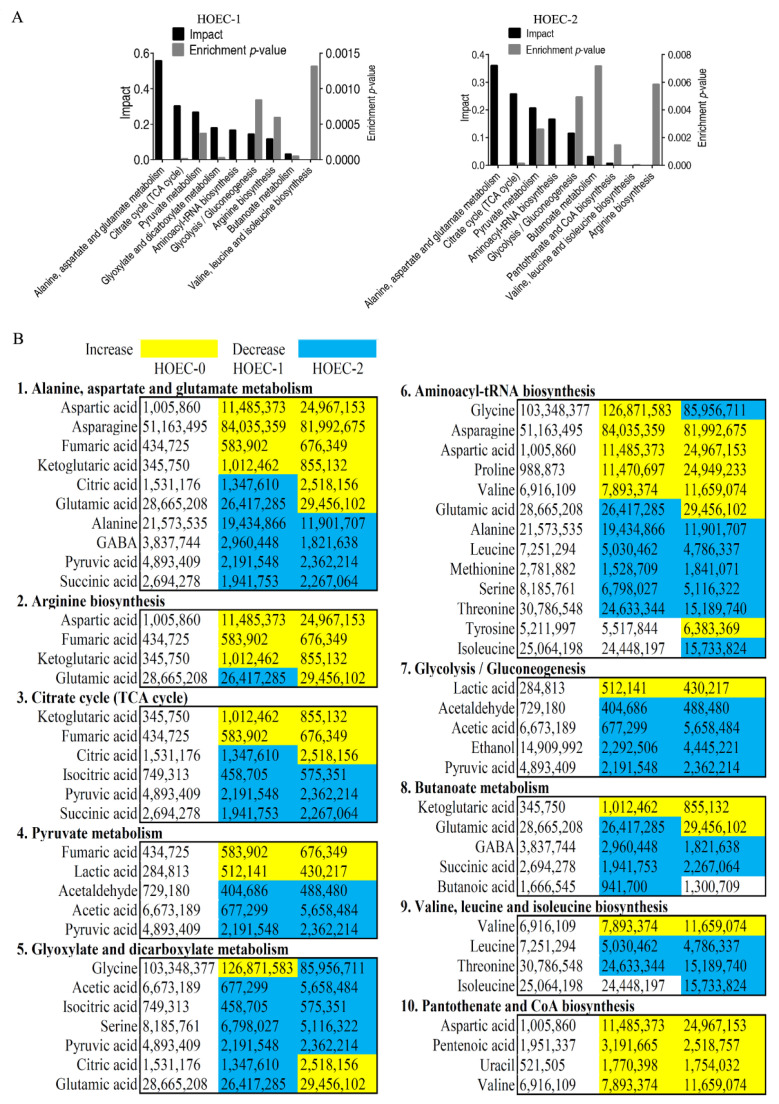
Pathway enrichment analysis of differential metabolites in HOEC cells after cisplatin treatment. Significantly enriched metabolic pathways (**A**). The abundance of metabolites in the enriched metabolic pathway (**B**). HOEC_0, HOEC_1 and HOEC_2 represent the HOEC cells treated with cisplatin for 0 h, 24 h or 48 h.

Similarly, the metabolomes of cisplatin treated or untreated HOEC cells were profiled by GC-MS (Figure 5A,B). Cluster analysis of all metabolites showed that the groups treated for 24 h or 48 h clustered together and separated from the untreated group (Figure 5C). Compared with untreated cells, 48 or 43 differential metabolites were identified in HOEC cells treated with cisplatin for 24 h or 48 h. Cluster analysis of the differential metabolites demonstrated that HOEC cells treated with cisplatin for 24 h or 48 h were isolated from untreated HOEC cells (Figure 6A). Compared with untreated HOEC cells, the Z-value map of HOEC cells treated with cisplatin for 24 h or 48 h showed 15 identical up-regulated and 19 identical down-regulated differential metabolites (Figure 6B). Differential metabolites at each time point of cisplatin exposure can be enriched into 9 metabolic pathways, of which 8 metabolic pathways were the same in 24 h or 48 h treatment cells (Figure 7A,B). Alanine, aspartate, and glutamate metabolism; citrate cycle and pyruvate metabolism were the three most important metabolic pathways (Figure 7A).

**Figure 8:**
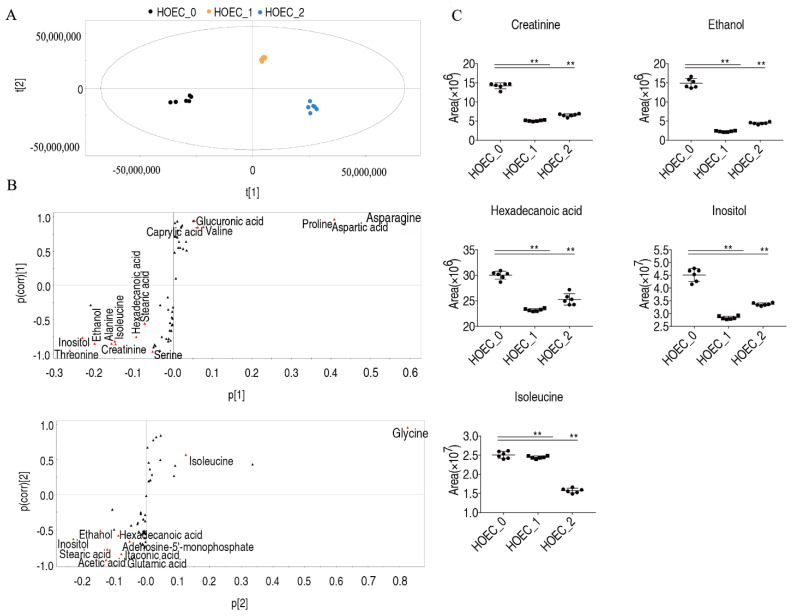
Screening of metabolic biomarkers in response to cisplatin exposure in HOEC cells. OPLS-DA analysis (**A**). S-plot analysis (**B**). Relative abundance of biomarkers (**C**). HOEC_0, HOEC_1 and HOEC_2 represent the HOEC cells treated with cisplatin for 0 h, 24 h or 48 h. **, *p* < 0.01.

OPLS-DA analysis of differential metabolites showed that t [1] could distinguish untreated HOEC cells from 2 groups treated for 24 h or 48 h, and t [2] could distinguish cells treated for 24 h from the cells untreated or treated for 48 h (Figure 8A). Marker metabolites induced by cisplatin were determined through S-plot analysis, and 15 and 10 marker metabolites were identified by t [1] and t [2], respectively (Figure 8B). Inositol, ethanol, isoleucine, hexadecanoic acid, and creatinine were five common biomarkers screened by two factors (Figure 8C).

### 2.5. Differential Metabolic Responses of SCC-9 and HOEC Cells to Cisplatin

**Figure 9:**
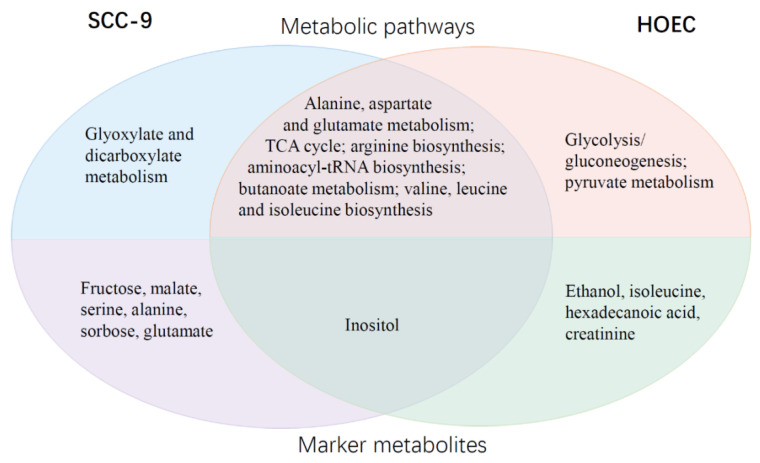
Comparisons of enriched metabolic pathways and marker metabolites of SCC-9 and HOEC cells in response to cisplatin stimulation.

We further analyzed the differential metabolic responses of oral cancer cells and normal cells to cisplatin by comparing the differences of enriched metabolic pathways and differential marker metabolites between the two cells. As mentioned above, differential metabolites were enriched in 7 metabolic pathways in SCC-9 cells and 8 metabolic pathways in HOEC cells, and 7 and 5 marker metabolites were identified in SCC-9 and HOEC cells, respectively. SCC-9 and HOEC cells shared six metabolic pathways, while glyoxylate and dicarboxylate metabolism pathway was unique to SCC-9 cells (Figure 9). Six SCC-9 cell specific marker metabolites were also found, including fructose, malate, serine, alanine, sorbose, and glutamate. The significance of these tumor cell-specific metabolic pathways or metabolites in the metabolic adaptation to cisplatin challenge and the development of drug resistance needs to be further studied. We noted that alanine, aspartate, and glutamate metabolism was the common enriched pathway in SCC-9 and HOEC cells, but alanine and glutamate were only identified as marker metabolites in SCC-9 cells. The weight difference of these two amino acids in each cell line may be a reasonable explanation. It seems that amino acid metabolism is more involved in the response of SCC-9 cells to cisplatin. These differences lay a foundation for tumor chemotherapy combined with metabolic intervention.

## 3. Discussion

Metabolic adaptation is conducive to the survival of tumor cells under drug challenge, but the metabolic pathways and corresponding marker metabolites leading to cisplatin resistance are not clear. Metabolomics analysis of drug-resistant cells can help us find the potential metabolic vulnerability of cisplatin resistant tumors [21]. For example, cisplatin resistant cells are more sensitive to nutritional deficiency than parental cisplatin sensitive cells. Cisplatin resistant cells are highly dependent on glutamine, and glutamine deprivation can re-sensitize them to cisplatin. Glutamine is mainly required for nucleotide biosynthesis in cisplatin resistant cells [21]. However, the early metabolic responses of oral cancer cells to cisplatin and the different responses of normal oral epithelial cells have not been well studied. In this study, we found that there were differences in the metabolic responses of oral cancer cells and normal oral epithelial cells to cisplatin, and we proposed several specific response metabolites and metabolic pathways of oral cancer cells.

Some studies tried to explore the early metabolic response of tumor cells to drugs. For example, short-term treatment of gastric cancer cells with 5-FU led to significant changes in cell metabolites, which was characterized by decreasing proline and increasing glutamate. This was due to the upregulation of proline dehydrogenase, which can promote the conversion of proline to glutamate [19]. The changes in these cells were also different from those observed in drug-resistant counterparts. Cisplatin/tamoxifen significantly reduced the level of phosphocholine in human breast cancer cells [22]. Anticancer drugs including cisplatin changed the glycine metabolism of lung cancer cells, which was characterized by inhibiting pyruvate metabolism and down-regulating betaine aldehyde and 5′-phosphoribosylglycinamide [23]. Metabolomics profiling revealed that when three-negative breast cancer (TNBC) cells were exposed to cisplatin and doxorubicin, their metabolites of arginine and polyamine were changed [24]. Inhibition of ornithine decarboxylase that promotes polyamine synthesis made tumor cells sensitive to chemotherapy [24,25]. Metabolomics analysis of mouse tissues was also used to study the effect of cisplatin exposure on metabolism in vivo [26]. Acute (short-term) cisplatin exposure increased the intra-tumoral level of S-methyl-5-thioadenosine (MTA) precursors in HNSCC bearing mice, and acquisition of cisplatin resistance also developed cross-resistance to ferroptosis agonists [12], suggesting that cisplatin can induce oxidative stress in tumor cells. Metabolomics profiling of tumor cells exposed to anticancer drugs is of great value for evaluating drug-induced metabolic response and drug efficacy, determining new therapeutic targets and screening possible noninvasive diagnostic markers [20,27]. Early metabolic response may provide some important information for evaluating the contribution of metabolic changes to the development of drug resistance and identifying the potential metabolic vulnerability for metabolic intervention in tumor therapy.

The metabolic mechanism of drug resistance is very complex. The acquisition of cisplatin resistance usually involves the adaptation of many metabolic pathways [2]. In HNSCC cells exposed to cisplatin for a short time, the differentially regulated pathways are somewhat similar to those previously found in chemoresistant tumor cells, but they also show some specificity. It was reported that HNSCC cells will produce cross-resistance to ferroptosis inducers, while acquiring cisplatin resistance, indicating the metabolic recovery of the cells from oxidative stress [12]. Integrated analysis of metabolomics and gene expression data indicated that the differentially regulated pathways in the cisplatin resistant cells centered on small molecule, amino acid and fatty acid catabolism and anabolism. The ^13^C flux experiments revealed that the catabolism and synthesis rates of serine and aspartate in cisplatin resistant cells drastically changed. This is consistent with the reprogramming of amino acid metabolism and increased serine production in this study.

The difference in the metabolic response to cisplatin between normal cells and tumor cells is not clear. The discovery of tumor cell-specific drug-induced metabolites is expected to provide a new target for tumor metabolic intervention. It was reported that the glucose and lipid metabolism of breast cancer cells and normal breast epithelial cells underwent differential reprogramming upon the treatment of triterpenic acids, plant-derived bioactive compounds [28]. In the current study, we found oral cancer cells and normal oral epithelial cells showed differential metabolic responses to cisplatin exposure. Glyoxylate and dicarboxylate metabolism was considered as cancer specific metabolic pathway, while fructose, malate, serine, alanine, sorbose, and glutamate were identified as specific response metabolites of cancer cells. The underlying mechanisms of these changes need to be further studied.

In addition, an important issue is whether these differences are intrinsic and independent of cisplatin treatment. It is well known that cancer cells undergo metabolic reprogramming. In our previous study, we found several differential metabolites between SCC-9 and HOEC cells [20]. In the current study, we first analyzed the metabolic changes (metabolic responses) of each cell line after exposure to cisplatin, and then compared the difference of their metabolic changes (differential metabolic responses), rather than directly analyzing metabolic differences of two cells exposed to cisplatin. That is, we focused on their differential response, regardless of the initial metabolic differences, even if they already existed. It is precisely because there are many metabolic differences between cancer cells and normal cells that we expect them to differentially respond to cisplatin.

We noted that the serine levels gradually increased in SCC-9 cells stimulated by cisplatin for 24 h and 48 h. Serine is a protein amino acid and substrate of One-Carbon Units (OCU). The increase of serine may facilitate the conversion of serine to glycine and OCU so as to promote the glutathione biosynthesis for anti-oxidation and provide carbon sources for nucleotide biosynthesis. In some tumors, the activation of de novo serine/glycine biosynthesis is considered to be the main factor of tumor pathogenesis, poor prognosis, and treatment resistance. Inhibition of the de novo serine/glycine biosynthesis pathway provides a promising strategy for tumor therapy [29]. Inhibition of serine metabolism decreased H3K4 tri-methylation and promoted cisplatin resistance in gastric cancer [30]. Phosphoglycerate dehydrogenase (PHGDH) is the rate-limiting enzyme in de novo serine synthesis. PHGDH knockout or inhibition slowed down cell proliferation, and then promoted drug resistance of tumor cells to chemotherapy [31,32]. The expression level of PHGDH was correlated with the clinicopathological features of bladder cancer, and the prognosis of patients with high PHGDH expression was poor. Compared with single drug, PHGDH inhibitor combined with gemcitabine/cisplatin can achieve synergistic tumor inhibition [32]. The mechanism and the significance of cisplatin-induced early serine elevation in oral cancer cells deserve further study.

## 4. Materials and Methods

### 4.1. Cell Culture and Treatment

The study involved immortalized normal human oral epithelial cell HOEC (BNCC340217) provided by BeNa Culture Collection (Bnbio, Beijing, China) and human tongue squamous cell carcinoma cell SCC-9 provided by Shanghai Guandao Biological Engineering Co., Ltd. (Sgdbio, Shanghai, China) [20]. Cells were maintained in medium DMEM with 10% FBS (GIBCO, Brooklyn, NY, USA), 100 U/mL penicillin G and 100 μg/mL streptomycin at 37 °C in a humidified atmosphere of 5% CO_2_. Cells cultured to 70% confluence in 100 mm petri dish were supplemented with 10.0 μM cisplatin (Sigma-Aldrich, Saint Louis, MO, USA) at approximately 20% maximal inhibitory concentration (IC_20_). The cells were further cultured at 37 °C for 24 h or 48 h, and then the cells were harvested for metabolomics analysis. Four replicates were prepared for each sample, and cells in the control groups were treated with the same amount of DMSO.

### 4.2. Cell Viability Assay

For cell viability assay, HOEC and SCC-9 cells in logarithmic growth were seeded in a 96-well plate at a concentration of 1 × 10^4^ cells/well. The cells were cultured for 24 h and then cisplatin solution was added at final concentrations of 50.0, 25.0, 12.5, 6.3, and 3.1 μM, respectively. Triplicates were employed at each concentration, and the medium containing the same amount of DMSO was used as the control. The cells were cultured at 37 °C in a 5% CO_2_ incubator for 48 h. The medium was replaced with 100 μL fresh DMEM containing 10 μL CCK8 solution (MedChemExpress, Monmouth Junction, NJ, USA), and cells were incubated at 37 °C for 2 h. Absorbance at 450 nm was measured by a microplate reader (Promega, Madison, WI, USA). The cell viability and the IC_50_ (50% growth inhibitory concentration) were determined according to the readings of the treated wells and the control wells (absence of test compound) with correction of background absorbance [23].

### 4.3. Gas Chromatography-Mass Spectrometry

Gas chromatography-mass spectrometry (GC-MS) was used to analyze the metabolic responses of SCC-9 and HOEC cells to cisplatin as described in previous study [20]. Briefly, the metabolites were extracted with methanol and derivatized by incubation in methoxyamine pyridine solution (20 mg/mL) at 37 °C for 120 min and in N,O-Bis (trimethylsilyl) trifluoroacetamide (TMSTFA) with 1% trimethylchlorosilane (TMCS) at 37 °C for 30 min. The derivatized samples were analyzed by Agilent 7890A GC equipped with an Agilent 5975C VL MSD detector (Agilent Technologies, Santa Clara, CA, USA). The relative peak area of internal standard ribitol was used as a reference to calculate the abundance of metabolites

### 4.4. Bioinformatics Analysis

Analysis of variance (ANOVA, α = 0.01) was used to compare the differences of metabolites between the two groups. A multivariate statistical analysis was performed via the MetaboAnalyst online Web site (www.metaboanalyst.ca, accessed on 25 January 2021) [33,34,35]. A Z-score analysis scaled each metabolite according to the reference distribution. Hierarchical cluster analysis (HCA), orthogonal partial least squares-discriminant analysis (OPLS-DA), and metabolite pathway enrichment analysis were performed as described in previous studies [20].

## Figures and Tables

**Table 1:** The common differential metabolites in SCC-9 cells treated with cisplatin for 24 h or 48 h.

Up-Regulated	Down-Regulated
malate, glucose, fructose, sorbose, fumaric acid, serine, benzoic acid, succinic acid, valine, leucine, threonine, citric acid, actate, lauric acid, hexadecanedioic acid	pyruvate, inosine, sebacic acid, hexanedioic acid, creatinine, adenosine, inositol, ornithine, alanine, AMP

## Data Availability

The data presented in this study are available in the article.
